# Congestion as a crucial factor determining albuminuria in patients with cardiorenal disease

**DOI:** 10.1093/ckj/sfae140

**Published:** 2024-05-15

**Authors:** Pau Llàcer, Marta Cobo Marcos, Rafael de la Espriella, Jara Gayán Ordás, Isabel Zegri, Aleix Fort, Adriana Rodríguez Chavarri, Ana Méndez, Zorba Blázquez, Pedro Caravaca Pérez, Jorge Rubio Gracia, Cristina Fernández, Alejandro Recio-Mayoral, Antonia Pomares, Jose Manuel García Pinilla, Jorge Vazquez López-Ibor, Almudena Castro, Maria Jose Soler, Jose Luis Górriz, Ramón Bascompte Claret, Paula Fluvià, Luis Manzano, Julio Núñez

**Affiliations:** Internal Medicine Department, Hospital Universitario Ramón y Cajal, IRYCIS, Madrid, Spain; Department of Medicine and Medical Specialties, Facultad de Medicina y Ciencias de la Salud, Universidad de Alcalá, Madrid, Spain; Department of Cardiology, Hospital Universitario Puerta de Hierro Majadahonda, Madrid, Spain; Centro de Investigación Biomédica en Red en Enfermedades Cardiovasculares, Madrid, Spain; Department of Cardiology, Hospital Clínico Universitario de Valencia, Valencia, Spain; Department of Cardiology, Hospital Universitario Arnau de Vilanova, Institut de Recerca Biomèdica de Lleida, Lleida, Spain; Department of Cardiology, Hospital de la Santa Creu i Sant Pau, Barcelona, Spain; Department of Cardiology, Hospital Universitari Dr Josep Trueta, Girona, Spain; Department of Cardiology, Hospital Universitario La Paz, Madrid, Spain; Department of Cardiology, Hospital Universitario Vall d’Hebron, Barcelona, Spain; Department of Cardiology, Hospital Universtiario Gregorio Marañón, Madrid, Spain; Department of Cardiology, Hospital Universitario Doce de Octubre, Madrid, Spain; Department of Internal Medicine, Hospital Universitario Lozano Blesa, University of Zaragoza, Zaragoza, Spain; Internal Medicine Department, Hospital Universitario Ramón y Cajal, IRYCIS, Madrid, Spain; Department of Medicine and Medical Specialties, Facultad de Medicina y Ciencias de la Salud, Universidad de Alcalá, Madrid, Spain; Department of Cardiology, Hospital Universitario Virgen Macarena, Sevilla, Spain; Department of Cardiology, Hospital de la Santa Creu i Sant Pau, Barcelona, Spain; Department of Cardiology, Hospital Universitario Virgen de la Victoria, Málaga, Spain; Department of Cardiology, Hospital Universitario Puerta de Hierro Majadahonda, Madrid, Spain; Department of Cardiology, Hospital Universitario La Paz, Madrid, Spain; Department of Nephrology, Hospital Universitario Vall d´Hebron, Barcelona, Spain; Department of Nephrology, Hospital Clínico Universitario Valencia, University of Valencia, Valencia, Spain; Department of Cardiology, Hospital Universitario Arnau de Vilanova, Institut de Recerca Biomèdica de Lleida, Lleida, Spain; Department of Cardiology, Hospital Universitari Dr Josep Trueta, Girona, Spain; Internal Medicine Department, Hospital Universitario Ramón y Cajal, IRYCIS, Madrid, Spain; Department of Medicine and Medical Specialties, Facultad de Medicina y Ciencias de la Salud, Universidad de Alcalá, Madrid, Spain; Centro de Investigación Biomédica en Red en Enfermedades Cardiovasculares, Madrid, Spain; Department of Cardiology, Hospital Clínico Universitario de Valencia, Valencia, Spain

**Keywords:** albuminuria, cardiorenal disease, congestive heart failure, congestion, predictors

## Abstract

**Background:**

Albuminuria could potentially emerge as a novel marker of congestion in acute heart failure. However, the current evidence linking albuminuria and congestion in patients with congestive heart failure (CHF) remains somewhat scarce. This study aimed to evaluate the prevalence of albuminuria in a cohort of patients with CHF, identify the independent factors associated with albuminuria and analyse the correlation with different congestion parameters.

**Methods:**

This is a subanalysis of the Spanish Cardiorenal Registry, in which we enrolled 864 outpatients with heart failure and a value of urinary albumin:creatinine ratio (UACR) at the first visit.

**Results:**

The median age was 74 years, 549 (63.5%) were male and 438 (50.7%) had a reduced left ventricular ejection fraction. A total of 350 patients (40.5%) had albuminuria. Among these patients, 386 (33.1%) had a UACR of 30–300 mg/g and 64 (7.4%) had a UACR >300 mg/g. In order of importance, the independent variables associated with higher UACR were estimated glomerular filtration rate determined by the Chronic Kidney Disease Epidemiology Collaboration equation (*R*^2^ = 57.6%), systolic blood pressure (*R*^2^ = 21.1%), previous furosemide equivalent dose (FED; *R*^2^ = 7.5%), antigen carbohydrate 125 (CA125; *R*^2^ = 6.1%), diabetes mellitus (*R*^2^ = 5.6%) and oedema (*R*^2^ = 1.9%). The combined influence of oedema, elevated CA125 levels and the FED accounted for 15.5% of the model's variability.

**Conclusions:**

In patients with chronic stable heart failure, the prevalence of albuminuria is high. The risk factors of albuminuria in this population are chronic kidney disease and hypertension. Congestion parameters are also associated with increased albuminuria.

KEY LEARNING POINTS **What was known:**

- Albuminuria could potentially emerge as a novel marker of congestion in acute heart failure. Nevertheless, the existing evidence connecting albuminuria and congestion in patients with congestive heart failure (CHF) remains somewhat limited.

**This study adds:**

- Among individuals with a diagnosis of chronic stable heart failure, a strikingly high prevalence of albuminuria is observed. The principal contributors to albuminuria in this population include chronic kidney disease and hypertension. It is essential to emphasize that markers of congestion are intricately associated with an increased likelihood of albuminuria in these cases.

**Potential impact:**

- When analysing a urinary albumin:creatinine ratio (UACR) test in patients with heart failure, it is crucial to consider their fluid overload status.
- In individuals experiencing fluid retention (wet patients), UACR levels may register higher compared with those who are not retaining excess fluids (dry patients).
- Future studies should explore the clinical insights that can be gleaned from observing UACR trajectories transitioning from wet to dry scenarios.

## INTRODUCTION

The presence of albuminuria has been of great interest in heart failure (HF) in recent years [1]. Chronic kidney disease (CKD) is defined according to the Kidney Disease: Improving Global Outcomes (KDIGO) guidelines by the estimated glomerular filtration rate (eGFR) and/or albuminuria [2]. Values of eGFR <60 ml/min/1.73 m^2^ and/or albuminuria [albumin excretion rate >30 mg/24 h or urinary albumin:creatinine ratio (UACR) >30 mg/g] establish the diagnosis of CKD. The prevalence of CKD in patients with congestive heart failure (CHF) is ≈70% according to a recently published study by our research group [3]. Albuminuria has been established as a risk factor for the development of cardiovascular diseases [4], including HF [5, 6]. Furthermore, it has been observed that albuminuria regression is associated with a decrease in cardiovascular risk [7]. It also has a prognostic value in established HF. Albuminuria is associated with increased mortality, increased risk of hospitalization [8] and more severe comorbidities [9].

Recent studies propose a potential link between albuminuria and the extent of congestion in patients with acute HF [10]. Congestion, recognized as the primary driver of HF readmissions, is advocated for comprehensive assessment, in line with consensus and clinical practice guidelines [11–14]. It is worth considering that albuminuria might serve as a novel marker of congestion in this context. Conversely, in patients with chronic stability, the existing evidence supporting the association between albuminuria and congestion is limited.

This study aimed to evaluate the prevalence of albuminuria in a cohort of patients with CHF, determine the independent factors associated with albuminuria and analyse the correlation with different congestion parameters.

## MATERIALS AND METHODS

### Study design and population

This is a subanalysis of the Spanish Cardiorenal Registry, where we prospectively evaluated a consecutive cohort of 1107 patients who attended a routine follow-up visit in 13 Spanish HF clinics, regardless of baseline eGFR, from October 2021 to February 2022 [3]. Diagnosis of HF was performed according to current European guidelines [15]. The only exclusion criterion was refusal to participate. This subanalysis only included patients with a UACR value at the first visit, a total of 864 patients. Data were collected on patient demographics, medical history, medical and device therapy at baseline, vital signs and physical examination, including oedemas. The oedema scoring system assigned points as follows: absent, 0 points; present in ankles, 1 point; present up to the knees, 2 points; and present up to the root of the limbs, 3 points.

This study complied with the Declaration of Helsinki and was approved by the local institutions’ ethics committees. Informed consent was obtained from all the subjects.

### Laboratory analysis

Blood and urine tests were assessed at baseline (within a 48-h window from inclusion) and analysed in the local laboratory of each participating site. eGFR was calculated from creatinine levels using the Chronic Kidney Disease Epidemiology Collaboration (CKD-EPI) equation and stratified according to KDIGO 2012 classification into four clinical strata: <30 ml/min/1.73 m^2^ (G4–G5), 30–44 ml/min/1.73 m^2^ (G3b), 45–59 ml/min/1.73 m^2^ (G3a) and ≥60 ml/min/1.73 m^2^ [2]. All patients had a prior eGFR assessment available in their medical chart for eGFR confirmation. Albuminuria was assessed in the first morning urine sample and stratified into three categories using UACR: A1 (normoalbuminuria), <30 mg/g; A2 (microalbuminuria), 30–300 mg/g; and A3 (macroalbuminuria), >300 mg/g.

### Statistical analysis

Continuous variables are presented as median [interquartile range (IQR)]. Categorical variables are expressed as percentages. Comparisons across albuminuria categories were performed by χ^2^ test for categorical variables. For continuous variables, one-way analysis of variance and the Kruskal–Wallis test were used for variables with a parametric and non-parametric distribution, respectively. The variables associated with albuminuria were evaluated by multivariate linear regression analysis. The contribution of the exposures to the proportion of the dependent variable variation was evaluated by *R*^2^. In the multivariable models, all variables listed in Table 1 were tested based on prior knowledge/biological plausibility, regardless of the *P*-value. We simultaneously tested the linearity assumption for all continuous variables and the variables were transformed using fractional polynomials when appropriate. Next, we derived a reduced and parsimonious model using backward stepwise selection on prior knowledge/biological plausibility, independent of the *P*-value. The covariates included in the final model were age, sex, systolic blood pressure (SBP), oedemas, basal eGFR (CKD-EPI), basal haemoglobin, antigen carbohydrate 125 (CA125) levels and basal furosemide equivalent dose (FED). The contribution of the covariates to the variability of UACR in the multiple linear regression model was assessed by the coefficient of determination (*R*^2^). We set a two-sided *P*-value of <.05 as the threshold for statistical significance. Stata 15.1 (version 15; StataCorp, College Station, TX, USA) was used for these analyses.

**Table 1:** Baseline characteristics.

	All sample	UACR <30 mg/g	UACR 30–300 mg/g	UACR >300 mg/g	
Variables	(*N* = 864)	[*n *= 514 (59.49%)]	[*n* = 286 (33.10%)]	[*n* = 64 (7.41%)]	*P*-value
Demographics and medical history					
Age (years), median (IQR)	74 (63–82)	72 (60–81)	77 (67–83)	75 (67.5–82.5)	<0.001
Male, *n* (%)	549 (63.54)	316 (61.44)	193 (67.48)	40 (62.5)	0.236
Hypertension, *n* (%)	599 (69.33)	316 (61.44)	224 (78.32)	59 (92.18)	<0.001
Diabetes mellitus, *n* (%)	346 (40.08)	169 (32.89)	133 (46.5)	45 (68.1)	<0.001
Dyslipidaemia, *n* (%)	518 (60.02)	284 (55.25)	192 (67.1)	42 (66.6)	0.002
Smoker, *n* (%)	80 (9.26)	45 (5.7)	29 (10.13)	6 (9.3)	0.947
Former smoker, *n* (%)	359 (41.55)	218 (42.41)	114 (39.86)	27 (42.18)	0.941
COPD, *n* (%)	155 (17.94)	92 (17.89)	50 (17.4)	13 (20.31)	0.867
Charlson comorbidity index, median (IQR)	5 (4–7)	5 (4–7)	6 (5–8)	7 (5–8)	<0.001
Atrial fibrillation, *n* (%)	453 (52.49)	259 (50.38)	162 (56.84)	32 (50)	0.199
Ischaemic aetiology, *n* (%)	262 (30.32)	192 (37.35)	113 (39.51)	29 (45.31)	0.438
Valvular heart disease, *n* (%)	171 (19.79)	86 (16.73)	69 (24.12)	16 (25)	0.023
ICD, *n* (%)	191 (22.13)	128 (24.9)	49 (17.13)	14 (21.87)	0.038
Resynchronization, *n* (%)	107 (12.38)	59 (11.47)	41 (14.33)	7 (10.93)	0.469
HF hospitalization in the previous year, *n* (%)	339 (39.24)	201 (39.10)	108 (37.76)	30 (46.87)	0.400
HF evolution (days), median (IQR)	720 (193–1888)	702 (179–1923)	739 (219–1810)	629 (159–2067)	0.553
Vital signs and basal assessment					
NYHA class, *n* (%)					0.059
I	126 (14.62)	93 (17)	36 (11.4)	5 (7.6)	
II	574 (66.59)	355 (65.1)	211 (67)	49 (74.2)	
III	160 (18.56)	96 (17.6)	64 (20.3)	12 (18.2)	
IV	2 (0.23)	0 (0)	2 (0.6)	0 (0)	
SBP (mmHg), median (IQR)	122 (108–136)	119 (105–131)	124 (110–140)	129 (114.5–150.5)	<0.001
DBP (mmHg), median (IQR)	69.79 (61–77)	70 (61–75)	70 (61–78)	70 (65–80)	0.0859
BMI (kg/m^2^), median (IQR)	27.25 (24–30)	27.34 (24.48–30)	26.95 (24.1–31.3)	27.59 (25–31.3)	0.349
Heart rate (bpm), median (IQR)	70.32 (61–77)	68 (60–75)	70 (61–78)	70 (65–81)	0.0273
Peripheral oedema, *n* (%)	192 (22.2)	94 (18.28)	75 (26.22)	23 (35.93)	<0.003
Echocardiography					
LVEF, *n* (%)					0.007
≥50	298 (34.49)	153 (29.76)	122 (42.65)	23 (35.93)	
40–50	142 (14.81)	82 (15.95)	35 (12.23)	11 , (17.18)	
≤40	438 (50.69)	279 (54.28)	129 (45.10)	23 (35.93)	
LVEF (%), median (IQR)	40 (30–55)	39 (30–53.1)	44 (32–56)	41 (33–56)	0.021
LVH, *n* (%)	422 (49.82)	235 (46.35)	145 (52.34)	42 (66.6)	0.010
TAPSE (mm), median (IQR)	19 (16–21)	19 (16–21)	19 (15–21.3)	19 (15–21)	0.895
sPAP (mmHg), median (IQR)	40 (31–52)	37 (29–50)	45 (35–55)	44 (35–54)	<0.001
Inferior cava vein (mm), median (IQR)	16 (14–20)	16 (14–19)	17 (13–21)	16 (14–20)	0.081
Lung B lines, *n* (%)	241 (35.86)	135 (33.41)	89 (40.63)	17 (34.69)	0.197
Laboratory data					
Creatinine (mg/dl), median (IQR)	1.34 (0.92–1.59)	1.12 (0.88–1.45)	1.32 (1–1.71)	1.62 (1.135–2.165)	<0.001
eGFR (ml/min/1.73 m^2^), median (IQR)	54.72 (37.52–76.87)	60 (41.75–82.6)	49.1 (34.68–68.1)	34.2 (23.66–54.87	<0.001
Urea (mg/dl), median (IQR)	59.85 (42–81.75)	53 (39–72)	66 (49–92)	77.5 (61–107)	<0.001
Sodium (mEq/l), median (IQR)	140 (138.4–142)	140 (139–142)	140 (138–142)	140 (138–142)	0.232
Potassium (mEq/l), median (IQR)	4.54 (4.2–4.9)	4.5 (4.2–4.9)	4.5 (4–4.83)	4.7 (4.3–5.07)	0.010
Potassium ≥5 mEq/l, *n*(%)	162 (18.75)	97 (17.8)	57 (18)	19 (28.8)	0.093
Potassium ≥5.5 mEq/l, *n* (%)	39 (4.51)	22 (4)	13 (4.5)	4 (6.2)	0.460
Chloride (mEq/l), median (IQR)	103 (100–106)	103 (101–106)	102 (99–105)	104 (101–106)	0.020
Haemoglobin (g/dl), median (IQR)	13.7 (12.2–15)	13.9 (12.6–15.2)	13.3 (11.7–14.9)	12.9 (11.85–14.2)	<0.001
Anaemia, *n* (%)	271 (31.37)	129 (25.09)	114 (39.86)	28 (43.75)	<0.001
Iron deficiency, *n* (%)	340 (42.24)	199 (41.63)	116 (43.44)	25 (41.66)	0.887
NT-proBNP (pg/ml), median (IQR)	1378.5 (575–3134)	992 (402–2464)	1969 (882–3721)	3079 (1044–7518)	<0.001
CA125 (U/ml), median (IQR)	14.7 (9.27–28)	13 (8.7–22.4)	18 (10–40)	18.4 (11.9–50.5)	<0.001
Uric acid (g/dl), median (IQR)	6.4(5–8)	6 (4.8–7.5)	6.6 (5.3–8.4)	7.7 (6.1–8.8)	<0.001
Glycosylated haemoglobin (%), median (IQR)	6 (5.5–6.5)	5.9 (5.5–6.4)	6 (5.5–6.6)	6.3 (5.75–7.4)	0.001
Total proteins (g/dl), median (IQR)	7 (6.6–7.29)	7 (6.6–7.3)	6.96 (6.6–7.28)	7 (6.5–7.2)	0.434
Albumin (g/dl), median (IQR)	4.18 (3.86–4.4)	4.2 (3.9–4.49)	4.1 (3.8–4.4)	3.94 (3.7–4.2)	<0.001
Total cholesterol (mg/dl), median (IQR)	148 (123–179)	150 (125–183)	144 (119–178)	146 (120–169.5)	0.106
Phosphorus (mg/dl), median (IQR)	3.5 (3.1–3.9)	3.5 (3.1–3.9)	3.5 (3.03–3.9)	3.7 (3.39–4.2)	0.004
iPTH (pg/ml), median (IQR)	77 (38–126)	74 (40–114)	74.6 (22.5–130)	128 (86–191)	<0.001
Treatment					
ACEI or ARB, *n* (%)	252 (29.2)	149 (28.29)	79 (27.71)	24 (37.5)	0.294
Sacubitril valsartan, *n* (%)	420 (48.61)	271 (52.72)	125 (43.70)	24 (37.5)	0.009
Beta-blocker, *n* (%)	681 (78.82)	415 (80.73)	217 (75.87)	49 (76.56)	0.245
MRA, *n* (%)	500 (57.94)	337 (65.69)	141(49.30)	22(34.37)	<0.001
SGLT2i, *n* (%)	511 (59.21)	314 (61.08)	162 (56.84)	35 (54.68)	0.376
Furosemide, *n* (%)	615 (71.18)	327 (63.61)	228 (79.72)	60 (93.75)	<0.001
FED (mg/day), median (IQR)	40 (0–80)	40 (0–60)	40 (20–80)	60 (40–80)	<0.001
Thiazides, *n* (%)	101 (11.76)	47 (9.19)	48 (16.84)	6 (9.5)	<0.005
IV iron therapy, *n* (%)	190 (22.1)	108 (21.17)	61 (21.40)	21(32.81)	0.100
ESA, *n* (%)	37 (4.29)	13 (2.52)	13 (4.5)	11 (17.46)	<0.001
VKa, *n* (%)	185 (21.54)	102 (19.96)	69 (24.29)	14 (21.87)	0.362
DOACs, *n* (%)	330 (38.55)	196 (38.50)	111 (39.22)	23 (35.93)	0.887
Digoxin, *n* (%)	54 (6.26)	25 (4.87)	24 (8.39)	5 (7.8)	0.125
Potassium binders, *n* (%)	47 (5.44)	26 (5)	15 (5.2)	6 (9)	0.351

COPD: chronic obstructive pulmonary disease; ICD: implantable cardioverter-defibrillator; BMI: body mass index; NYHA, New York Heart Association; LVH: left ventricular hypertrophy; TAPSE: tricuspid annular plane systolic excursion; sPAP: systolic pulmonary artery pressure; iPTH: intact parathyroid hormone; ACEi: angiotensin-converting enzyme inhibitor; ARB: angiotensin II receptor blocker; MRA: mineralocorticoids receptor antagonist; SLGT2i: sodium–glucose co-transporter 2 inhibitor; IV: intravenous; ESA: erythropoiesis-stimulating agent; VKa: vitamin K antagonist; DOACs: direct oral anticoagulants.

The following variables only have the indicated values available: sPAP (529), inferior cava vein (674), lung B lines (672), NT-proBNP (780), uric acid (802), albumin (810) and total protein (820).

## RESULTS

The median age was 74 years (IQR 63–82), 549 (63.5%) were male and 438 (50.7%) had a reduced left ventricular ejection fraction (LVEF). The median creatinine and eGFR were 1.34 mg/dl (IQR 0.92–1.59) and 54.7 ml/min/1.73 m^2^ (IQR 37.52–76.87), respectively. The median of the amino-terminal fraction of the brain N-terminal pro b-type natriuretic peptide (NT-proBNP) and CA125 were 1378 pg/ml (IQR 575–3134) and 14.7 U/ml (IQR 9.27–28), respectively. The median UACR was 22 mg/g (IQR 7.7–64.9). A total of 350 patients (40.5%) had albuminuria. Among these patients, 386 (33.1%) had a UACR of 30–300 mg/g and 64 (7.4%) had a UACR >300 mg/g (Table 1).

### Baseline characteristics across UACR categories

The baseline characteristics of the study sample across UACR categories are presented in Table 1. Patients with an increased UACR were older and exhibited a more adverse risk profile (particularly among those exhibiting macroalbuminuria), characterized by a higher prevalence of hypertension, diabetes mellitus and dyslipidaemia than those with normal UACR. A higher Charlson Comorbidity Index was more common in patients with an elevated UACR. Regarding vital signs and physical examination, patients with elevated albuminuria had a higher systolic blood pressure (SBP) and heart rate and oedemas. In the echocardiographic findings, elevated LVEF and pulmonary artery pressure are prominent in the patients with elevated UACR. In patients with an elevated UACR, renal function was worse, and haemoglobin was lower than in those with normal UACR. The CA125 and NT-proBNP levels were higher when albuminuria was elevated, mainly in the macroalbuminuria group. In the macroalbuminuria group, the utilization of sacubitril–valsartan and mineralocorticoid receptor antagonists was less frequent, while the FED was notably higher.

### Factors associated with higher UACR

**Figure 1:**
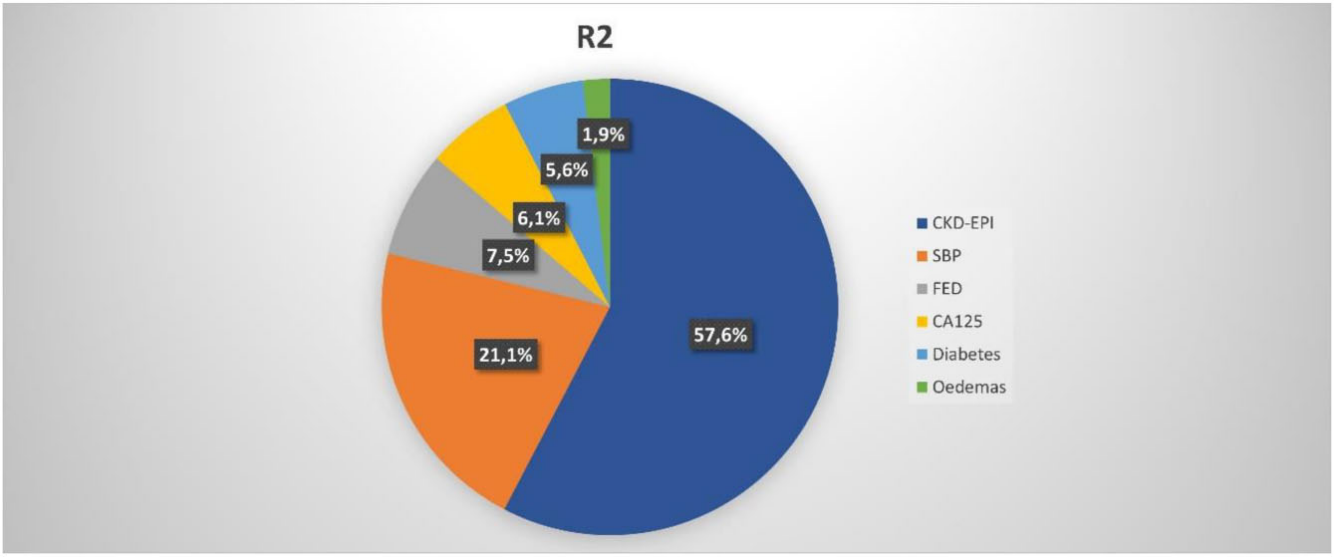
Predictors of UACR after multivariate analysis in order of importance assessed by the coefficient of determination (*R*^2^).

**Figure 2:**
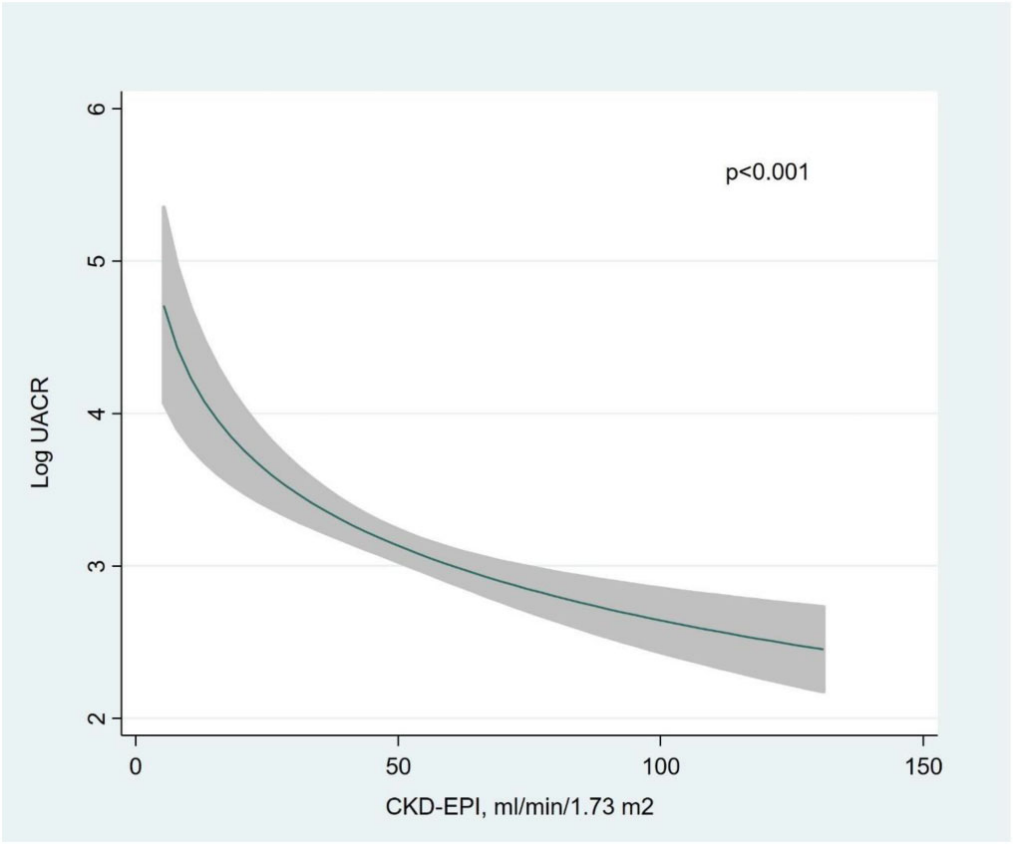
Relationship between eGFR (functional form) and UACR (logarithmic form) after multivariable analysis.

**Figure 3:**
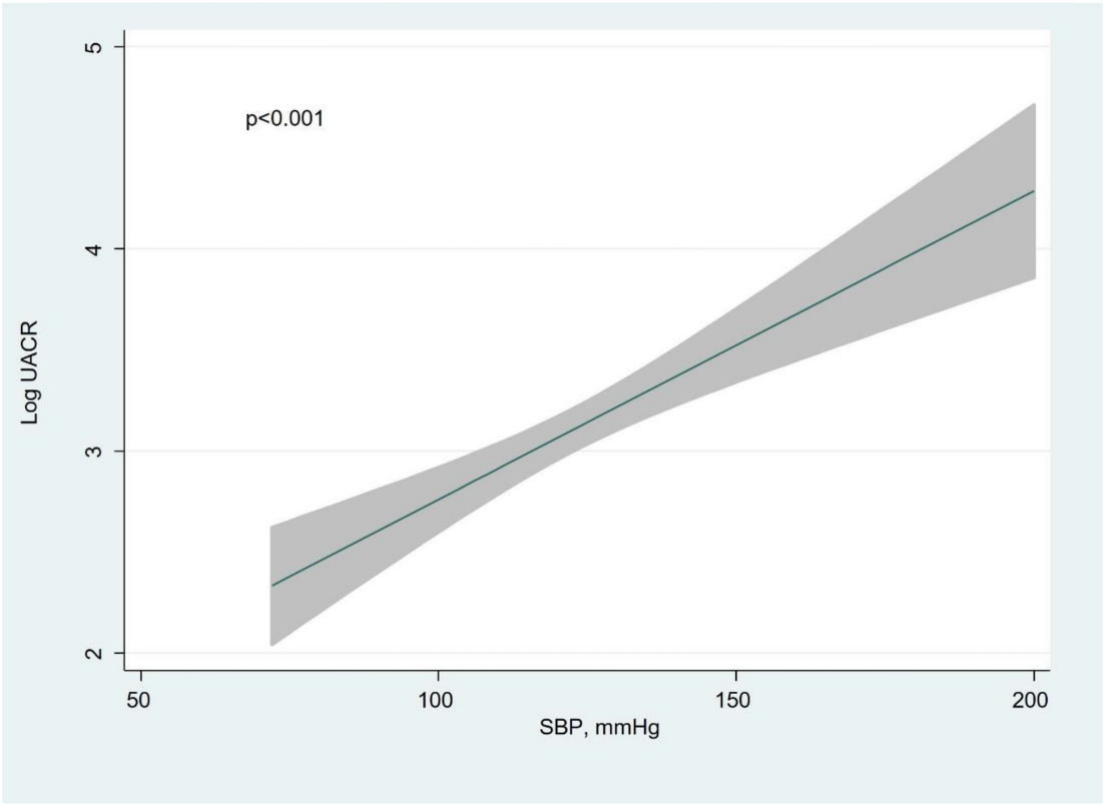
Relationship between SBP (functional form) and UACR (logarithmic form) after multivariable analysis.

**Figure 4:**
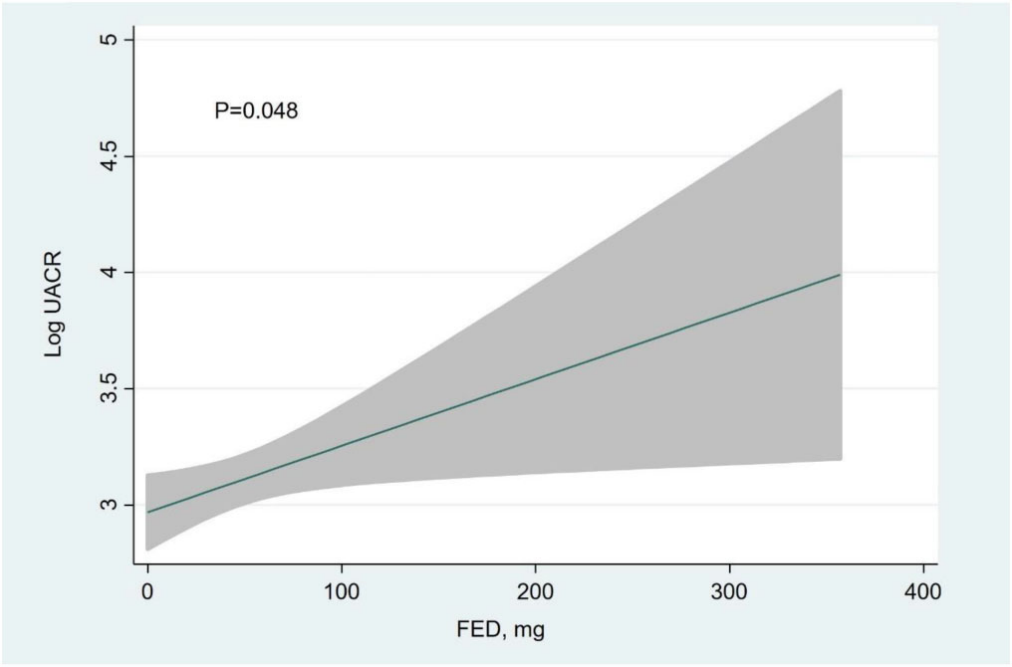
Relationship between FED (functional form) and UACR (logarithmic form) after multivariable analysis.

**Figure 5:**
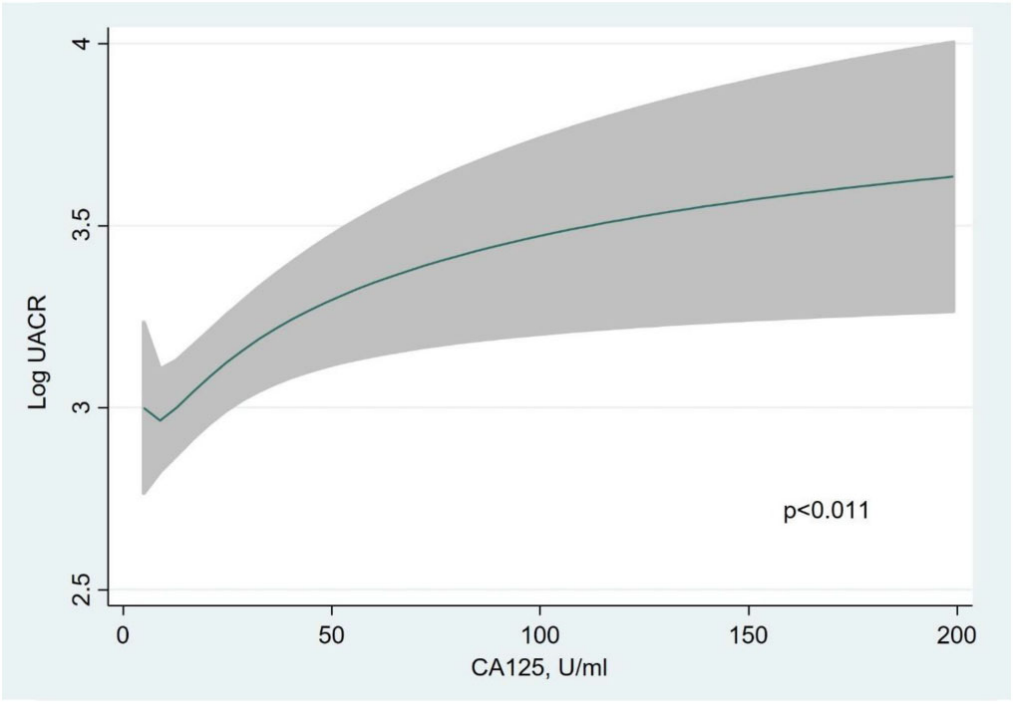
Relationship between CA125 (functional form) and UACR (logarithmic form) after multivariable analysis.

**Figure 6:**
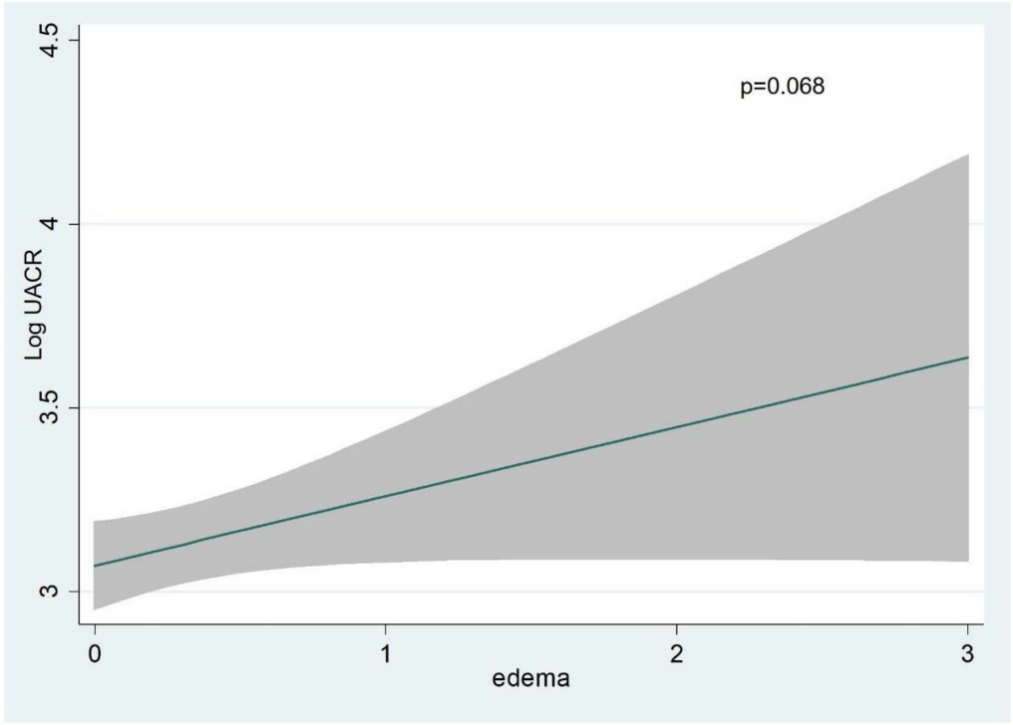
Relationship between oedemas (functional form) and UACR (logarithmic form) after multivariable analysis.

**Figure 7:**
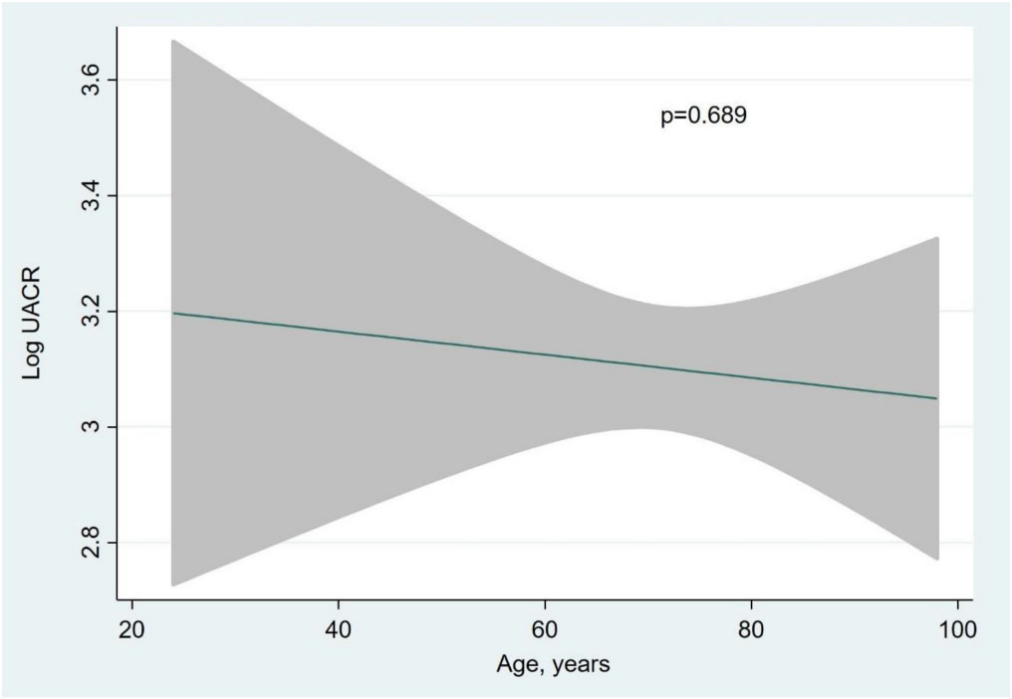
Relationship between age (functional form) and UACR (logarithmic form) after multivariable analysis.

Multivariate analysis revealed that independent variables associated with higher UACR (and explaining up to 95% of the model variability) (Fig. 1): eGFR showed an inverse and near-linear relationship (*R*^2^ = 57.6%, *P* < .001) (Fig. 2), whereas the association was positive and linear with SBP (*R*^2^ = 21.1%, *P* < .001) (Fig. 3). Other covariates associated with higher UACR were previous FED, CA125 and oedemas, all showing a positive and linear relationship with UACR (*R*^2^ = 7.5%, *P* < .048; *R*^2^ = 6.1%, *P* < .011; and *R*^2^ = 1.9%, *P* = .064, respectively) (Figs. 4–6) and previous diagnosis of diabetes mellitus (*R*^2^ = 5.6%, *P* < .001). Likewise, age (*P* = .689) (Fig. 7), sex (*P* = .575) and haemoglobin (*P* = .828) were not predictors of UACR. Overall, this multivariate model accounted for 17% of the variability in UACR.

### UACR and congestion parameters

The combined influence of oedema, elevated CA125 levels and FED—variables strongly associated with hydrosaline overload—accounted for 15.5% of the model's variability.

## DISCUSSION

In this study, which enrolled a cohort of 864 patients with CHF accompanied by an initial UACR determination, the main findings were a high prevalence of albuminuria among participants, eGFR and SBP emerged as the principal predictors for elevated UACR and congestion was independently associated with albuminuria in HF, even in a stable scenario.

The prevalence of albuminuria among HF patients, based on previous studies, typically falls within the range of 20–40% [1, 16, 17]. However, our study, which stands as the largest registry of patients with CHF that registered albuminuria, reflects a notably higher prevalence rate at 40.5%, mainly microalbuminuria. Consistent with findings in other studies, this increased prevalence was particularly notable among patients with HF with preserved ejection fraction. Interestingly, microalbuminuria was the most common presentation, although cases of macroalbuminuria were also observed [17, 18].

Patients with albuminuria were older and exhibited a less favourable risk profile. These patients displayed a higher likelihood of comorbidities such as hypertension, diabetes mellitus and dyslipidaemia compared with individuals with a normal UACR. Additionally, they more frequently presented with impaired renal function and more severe HF symptoms, including more pronounced signs of congestion.

In this cohort of patients, the group's prior study analysing the prevalence of CKD determined a prevalence rate of nearly 60% [3]. Furthermore, 13% of patients with eGFR ≥60 ml/min/1.7 m^2^ exhibited albuminuria.

### Predictors of elevated UACR

In our cohort, after multivariable analysis, the independent risk factors associated with albuminuria were the presence of CKD and high BP. Both explained >70% of the model. Other factors included diabetes, previous FED, CA125 and oedemas.

Albuminuria and its connection to HF is complex, with multiple underlying potential mechanisms. This association is primarily linked to impairment of the kidney filtration barrier, which includes damage to the endothelium and tubular structures and the presence of coexisting medical conditions such as hypertension, diabetes mellitus and obesity [1]. These alterations in the kidney's function trigger a cascade of events, resulting in systemic inflammation and activation of neurohormonal pathways, particularly the renin–angiotensin–aldosterone system (RAAS). This, in turn, results in volume overload due to enhanced retention of sodium and water, thereby fostering the onset and progression of HF [1].

In patients with HF and without diabetes, hypertension or CKD, albuminuria can often be attributed to HF itself [1]. In individuals with HF, elevated central venous pressure exerts pressure on the renal system, resulting in increased renal venous congestion. This congestion, in turn, leads to reduced renal perfusion pressure, ultimately causing a decrease in eGFR. To counteract these adverse changes, compensatory mechanisms are triggered, including activation of the RAAS [19].

A noteworthy percentage of patients displayed albuminuria despite the absence of hypertension, diabetes or CKD. This observation underscores the significant role played by HF itself and the associated congestion, even during the chronic stable phase. In our study, CA125 accounted for a larger proportion of the multivariate model's variance compared with diabetes mellitus (*R*^2^ = 6.1% versus 5.6%). These findings emphasize that HF, and the resulting congestion, can, to a considerable extent, account for the presence of albuminuria in our patient population.

### Congestion and UACR in CHF

In the present study, even in patients with chronic stable HF, congestion surrogates such as CA125, previous FED or oedemas played a relevant role as predictors of albuminuria. Other studies have examined the relationship of hydrosaline overload and albuminuria, but predominantly in acute scenarios, both new-onset and worsening HF. Boorsma *et al*. [10] observed in the BIOlogy Study to TAilored Treatment in Chronic Heart Failure cohort in CHF patients that congestion parameters such as biomarkers (including CA125), ultrasound and clinical parameters (oedema) were independently associated with the presence of albuminuria after multiple regression analysis. The relationship of congestion parameters in the chronic setting has not been previously evaluated as in our cohort. In the multivariate analysis, CA125, oedema and previous FED were independent predictors of the presence of albuminuria.

Notably, this is a cohort of patients with cardiorenal syndrome with a prevalence of CKD >70%, and CA125 levels predicted the presence of albuminuria in a model that included renal function. As is well known, CA125 values are not influenced by renal function. Instead, CA125 levels exhibit a strong correlation with indicators of right-sided HF, which are frequently encountered in this specific patient population. Moreover, the established link between CA125 and renal congestion further underscores the significance of this finding [20, 21].

Generally, renal damage resulting from hydrosaline overload stems not only from intravascular renal congestion, but also from parenchymal congestion and the third space (renal tamponade) [22]. Therefore, a tissue congestion parameter (like oedema or CA125), which often aligns with intravascular congestion in most patients, would more accurately predict the onset of renal impairment associated with hydrosaline overload.

### Clinical implications

When interpreting a UACR test in patients with HF, it seems essential to account for fluid overload status. In wet patients, UACR may be higher than in dry patients. Further studies may evaluate the clinical information provided by UACR trajectories from wet to dry scenarios.

### Limitations

This study has several limitations. First, UACR was assessed only at baseline and not continuously measured during follow-up. Second, it is important to note that patients were recruited from specialized HF clinics. As a result, any extrapolation of these findings to other follow-up methods, healthcare systems or countries should be interpreted with caution. Third, other ultrasound parameters of congestion, such as renal venous flow patterns or suprahepatic vein flow, were not available and there were missing values in the inferior vena cava and B lines to incorporate into the final model. Fourth, bioimpedance analysis was not done for volume assessment. Fifth, we do not have obesity data and thus were unable to analyse it. Sixth, the multivariate model showed a limited ability to predict albuminuria variability. This issue may reflect an incomplete understanding of albuminuria in cardiorenal patients and the lack of assessment of well-known factors related to the severity of albuminuria. And finally, as an observational study, there are several unmeasured confounders that may be operating or even modifying the current findings.

## CONCLUSIONS

In patients with chronic stable HF, the prevalence of albuminuria is high. The risk factors of albuminuria in this population are CKD and hypertension. Congestion parameters are also associated with increased albuminuria.

## Data Availability

The data underlying this article will be shared upon reasonable request to the corresponding author.
